# An Injectable Nano-Enabled Thermogel to Attain Controlled Delivery of p11 Peptide for the Potential Treatment of Ocular Angiogenic Disorders of the Posterior Segment

**DOI:** 10.3390/pharmaceutics13020176

**Published:** 2021-01-28

**Authors:** Lisa Claire du Toit, Yahya Essop Choonara, Viness Pillay

**Affiliations:** Wits Advanced Drug Delivery Platform Research Unit, Department of Pharmacy and Pharmacology, School of Therapeutic Sciences, Faculty of Health Sciences, University of the Witwatersrand, Johannesburg, 7 York Road, Parktown 2193, South Africa; lisa.dutoit@wits.ac.za (L.C.d.T.); viness.pillay@wits.ac.za (V.P.)

**Keywords:** angiogenesis, peptide, ocular drug delivery, thermosensitive hydrogel, nanoparticles

## Abstract

This investigation focused on the design of an injectable nano-enabled thermogel (nano-thermogel) system to attain controlled delivery of p11 anti-angiogenic peptide for proposed effective prevention of neovascularisation and to overcome the drawbacks of the existing treatment approaches for ocular disorders characterised by angiogenesis, which employ multiple intravitreal injections of anti-vascular endothelial growth factor (anti-VEGF) antibodies. Synthesis of a polyethylene glycol-polycaprolactone-polyethylene glycol (PEG-PCL-PEG) triblock co-polymer was undertaken, followed by characterisation employing Fourier-transform infrared (FTIR) spectroscopy, nuclear magnetic resonance (NMR) spectroscopy and differential scanning calorimetry (DSC) to ascertain the chemical stability and integrity of the co-polymer instituted for nano-thermogel formulation. The p11 anti-angiogenic peptide underwent encapsulation within poly(lactic-*co*-glycolic acid) (PLGA) nanoparticles via a double emulsion solvent evaporation method and was incorporated into the thermogel following characterisation by scanning electron microscopy (SEM), zeta size and zeta-potential analysis. The tube inversion approach and rheological analysis were employed to ascertain the thermo-sensitive sol-gel conversion of the nano-thermogel system. Chromatographic assessment of the in vitro release of the peptide was performed, with stability confirmation via Tris-Tricine PAGE (Polyacrylamide Gel Electrophoresis). In vitro biocompatibility of the nano-thermogel system was investigated employing a retinal cell line (ARP-19). A nanoparticle size range of 100–200 nm and peptide loading efficiency of 67% was achieved. Sol-gel conversion of the nano-thermogel was observed between 32–45 °C. Release of the peptide in vitro was sustained, with maintenance of stability, for 60 days. Biocompatibility assessment highlighted 97–99% cell viability with non-haemolytic ability, which supports the potential applicability of the nano-thermogel system for extended delivery of peptide for ocular disorder treatment.

## 1. Introduction

Retinopathy of prematurity (RP), age-related macular degeneration (AMD) and diabetic retinopathy (DR) are angiogenesis-related ocular diseases, which are prevalent from youth to old age and are principally attributed to the occurrence of irreversible blindness globally [1,2,3]. Even though these diseases affect all age groups, the pathology of these diseases are similar and include abnormal neovascularisation. Growth of new blood vessels from the retina of DR and RP patients leads to bleeding inside the vitreous and ends in fibrosis and visual loss [4]. In AMD patients, complete loss of vision occurs due to the overgrowth of blood vessels over the retina from the choroid region [4]. The major reason behind neovascularisation is hypoxia and oxidative stress which further triggers a cascade of angiogenic events. Vascular Endothelial Growth Factor (VEGF) is one of the key components in the normal angiogenesis cascade [5]. During angiogenesis, the endothelial cells interact through integrin molecules in the extracellular matrix for subsequent endothelial cell activation, proliferation and migration [6].

Current treatment strategies are thus focused on VEGF inhibition using neutralising antibodies against VEGF, VEGF receptor-suppressing SiRNA (small interfering RNA) or by inhibiting VEGF signaling (kinase inhibitor). There are currently four FDA-approved anti-VEGF therapies for the treatment of AMD: Macugen^TM^ (pegaptanib sodium), Lucentis^TM^ (ranibizumab), Eylea™ (Aflibercept) and most recently, Beovu™ (Brolucizumab) [7]. However, there are concerns regarding the administration of anti-VEGF treatment since VEGF is normally expressed in the eye and also serves as a survival factor and neuroprotectant for retinal neurons [8,9,10]. Moreover, systemic administration of anti-VEGF therapy will also affect other ocular tissue and organ systems [11].

Partial regulation of angiogenesis is via specific integrin transmembrane glycoproteins, hence current research is focused largely on anti-integrin therapy [12,13]. High expression of integrin α_v_β_3_ occurs in angiogenic endothelial cells and tumours [12], with selective expression of integrin α_v_β_3_ on the choroid neovascularisation (CNV) membrane during ocular angiogenesis, whereas in DR tissue, there is the presence of both α_v_β_3_ and α_v_β_5_ [13]. A few investigations highlighted that integrin antagonists can be considered as a target for ocular angiogenic disorders as an emerging treatment approach. p11 is a cell permeable hexapeptide (HS-DVHK) with a SDV sequence specifically bound to α_v_β_3_ integrins instead of the RGD sequences, a common binding sequence of the integrin motif. p11 inhibits neovascularisation via α_v_β_3_ integrin blocking without any side effects [14]. Bang et al. [14] demonstrated that this results in the suppression of bFGF-induced human umbilical vein endothelial cell (HUVEC) proliferation in endothelial cells, achieved through disruption of mitogen-activated protein kinase (MEK) and extracellular signal-regulated kinase (ERK)1/2 signaling (through inhibition of their phosphorylation at 10 µg/mL) and p53-mediated induction of apoptosis (at 50 µg/mL). This suppression is thus dose dependent. Even though the mechanism of anti-angiogenesis of p11 peptide has been studied in detail, a delivery system for p11 peptide has not been established as yet. Small peptides are always advantageous as therapeutic agents because of their non-toxicity and high specificity. However, the delivery of the peptides, with maintenance of their integrity in a sustained manner, is a persistent challenge.

Substantial advancement in the field of ocular drug delivery has been witnessed through recent developments in pharmaceutical ocular formulation technology including cyclodextrins, polymeric gel and colloidal systems [15]. However, the route of administration to the ocular posterior segment is still a hurdle, and this is the reason that angiogenic ocular disorders are the foremost cause of blindness. Among the various routes of drug administration, oral and systemic delivery of anti-angiogenic drug/peptide is not suitable since it requires considerable drug loading and causes systemic side effects [16]. Intravitreal injection is the current FDA-approved therapy. This mainly bypasses both blood retinal barriers as well as structural and functional barriers. However, ocular diseases being chronic in nature require continuous and repeated injections, which can cause retinal detachment, endophthalmitis, cataract and haemorrhage, which affects patient compliance in repeated injections [17]. Non-degradable and biodegradable implants can be an effective way to ensure long-term sustainable and stable delivery of protein for the treatment of ocular diseases. However, the stability of proteins/peptides in prolonged polymer matrices until the time of delivery requires remarkable improvement. Recently, hydrogels have been studied for stable delivery of proteins and peptides and have the advantage of good biocompatibility [18]. Among different hydrogel systems, thermo-sensitive in situ gel-forming hydrogels have gained greater importance for protein delivery because of their feasibility for enabling incorporation of proteins at low temperature (solution) and gelling after injection at physiological temperature [19].

**Figure 1:**
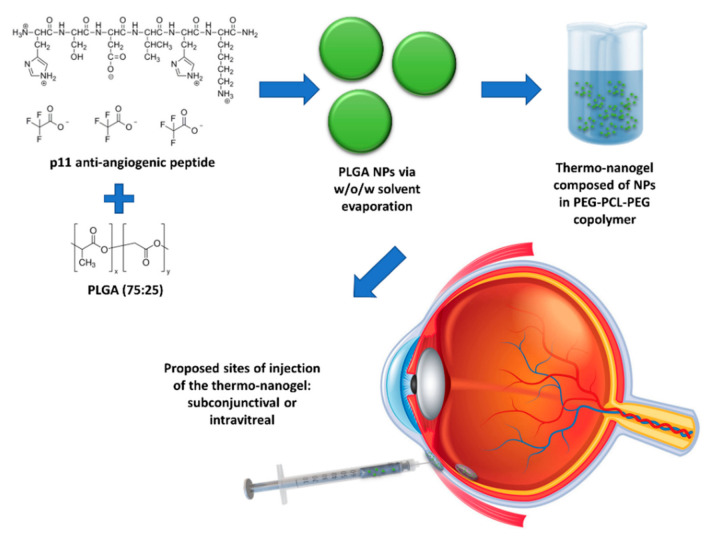
Schematic depiction of the componential formulation and proposed delivery of the thermo-nanogel system.

The current investigation is centred on the construction of a nanoparticle (NP)-embedded injectable thermosensitive copolymeric hydrogel system for effective ocular delivery of anti-angiogenic peptides in a sustainable manner without affecting their stability. p11 peptide was encapsulated in poly(lactic-*co*-glycolic acid*)* (PLGA) nanoparticles and embedded into an injectable polyethylene glycol-polycaprolactone-polyethylene glycol (PEG-PCL-PEG)-based thermogel system (nano-thermogel) for the proposed purpose of introduction into and gel formation in the eye, following injection either in the subconjunctival space or intravitreally (Figure 1). PLGA, a FDA-approved biodegradable polymer, has been applied for the delivery of various therapeutic agents in the eye and is generally well tolerated in vivo [20,21,22]. Moreover, several studies have been reported where PLGA nanoparticles mediated efficient and stable delivery of peptide molecules [23,24].

Various investigators have reported the synthesis of the biodegradable thermosensitive hydrogel based on the PEG-PCL-PEG and PCL-PEG-PCL triblock copolymers [18,25,26,27,28,29], which improves on the biodegradability of PCL through incorporation of molecular blocks of the hydrophilic PEG [29]. The hydrogel is a flowing sol at low temperature, that can be injected in combination with a pharmaceutical agent, forming a nonflowing gel at body temperature, thus serving as a sustained drug delivery site in vivo [18,25,26,27] and demonstrating potential ophthalmic applications [30].

The PEG-PCL-PEG triblock copolymer’s chemical structure and integrity was evaluated using FTIR and ^1^H-NMR spectroscopy. In addition to PEG-PCL-PEG, Pluronic F 127 (PLU) and a mixture of PEG-PCL-PEG/PLU hydrogels were prepared for a comparative evaluation. This approach enabled the development of a drug delivery system which has the potential for the stable delivery of anti-angiogenic p11 peptide to the retina over a prolonged period, thereby overcoming the limitations associated with repeated intravitreal injections. The thermosensitivity of the hydrogel was evaluated in conjunction with the release kinetics of the peptide over a period of 2 months. In vitro biocompatibility and haemolytic properties of the developed system were assessed in order to ascertain the safety of the drug delivery system.

## 2. Materials and Methods

### 2.1. Materials

ε-Caprolactone (ε-CL), poly(vinyl alcohol) (PVA MW 14KDa), methyl thiozol tetrazolium (MTT), poly(ethylene glycol) methyl ether (MPEG, MW = 4000), stannous octoate (Sn(Oct)_2_, Sigma, St Louis, MO, USA) isophorone diisocyanate (IPDI), Pluronic F 127 triblock copolymer, Tris-HCl, tricine, TEMED, ammonium per sulphate, 40% acrylamide-bis acrylamide solution, sodium dodecyl sulphate and ultra-low molecular weight protein marker were acquired from Sigma Aldrich (St Louis, MO, USA). Sodium azide was obtained from UniLab, Republic of South Africa. P11 hexapeptide (Sequence H-S-D-V-H-K, MW 721 Da) and FITC-p11 hexapeptide (Sequence FITC-Ah_x_-H-S-D-V-H-K, MW 1224 Da) were obtained from Peptorn Private LTD (Daejeon, Korea). PBS 1X was purchased from Merck (New York, NY, USA). Retinal pigment epithelial cells (RPE-1) were obtained from ATCC (Manassas, VA, USA). Dulbecco’s modified Minimum Essential Medium (MEM), Trypsin EDTA, FBS and penicillin/streptomycin solutions were purchased from Sigma Aldrich (St Louis, MO, USA). PLGA with monomer ratio (lactic acid and glycolic acid) of 75/25 was obtained as a gift from PURASORB, Holland. Tween^®^ 80, dichloromethane and petroleum ether were purchased from Merck (Darmstadt, Germany).

### 2.2. Preparation of PLGA Nanoparticles

A double emulsion solvent evaporation (water-in-oil-in-water, w/o/w) technique was employed for PLGA nanoparticle synthesis. Briefly, 20 mg PLGA (75/25) in 2 mL of dichloromethane was injected into 200 µL of PBS pH 7.4 containing 20 µg FITC–p11 peptide (w1/O) and agitated at 3000 rpm for 3 min using a magnetic stirrer. This emulsion was then injected into 10 mL of a second aqueous phase composed of 1.5% *w*/*v* PVA and 0.02% *w*/*v* Tween^®^ 80 and agitated at 6000 rpm over varying time periods (2, 3, 5, 8 and 10 min). Thus, five different nanoparticle formulations were prepared (Formulation 1, F 1, to Formulation 5, F 5). The emulsion was allowed to remain un-agitated for 2 min and then incubated on ice for 15 min. Thereafter, the organic solvent was evaporated using a rotavapor under vacuum with continuous agitation at 500 rpm (25 °C) for 3 h. The nanoparticles were centrifuged at 25,000 rpm for 5 min at 4 °C. The harvested nanoparticles were washed twice with deionised water and re-suspended in deionised water for lyophilisation. The lyophilised nanoparticles were stored at 4 °C until further use and analysis. The preferred formulation having a combination of comparatively small nanoparticle diameter, good polydispersity index (low) and enhanced encapsulation efficiency was identified for future characterisations.

### 2.3. Characterisation of the PLGA Nanoparticles

#### 2.3.1. Measurement of the PLGA Nanoparticle Size, Heterogeneity and Surface Stability

The diameter of the synthesised placebo and peptide-loaded PLGA nanoparticles were ascertained via dynamic light scattering (DTS nano, Malvern Instruments Ltd., Worcestershire, UK). Samples were re-suspended and diluted with deionised water and analysed. The mean diameter and poly-dispersity indices were calculated as a mean of three distinct batches for each formulation. The zeta potential (ζ) of the nanoparticles was measured in water by means of a Zetasizer Nano Z (Malvern Instruments Ltd., Worcestershire, UK). An average of 3 measurements was taken as the data point.

#### 2.3.2. Determination of Nanoparticle Morphology

Scanning electron microscopic images (FEI Nova Nanolab 600 FIB/SEM, Hillsboro, OR, USA) were acquired for the analysis of the morphology of the nanoparticles. Dilute preparations of nanoparticles were dispersed on aluminium stubs, dehydrated by air drying and sputter coated with gold. Nanoparticles encapsulating FITC-conjugated p11 peptide were further imaged using confocal microscopy (CF5-Zeiss LSM 510, Oberkochen, Germany) using a 488 nm excitation filter for FITC.

#### 2.3.3. Determination of Nanoparticle Encapsulation Efficiency

Peptide encapsulation efficiency of the nanoparticles was analysed after separation of the particle from aqueous preparation followed by solvent evaporation. As described by Geng et al. [31], the quantity of free peptide present in the supernatant aqueous phase after separation of the particle by centrifugation at 20,000 rpm for 5 min was determined by ultra- performance liquid chromatography (UPLC, Waters^®^ Acquity UPLC^TM^ system, Miliford, MA, USA). Triplicate experiments were conducted for peptide extraction and analysis for each formulation. The encapsulation efficiency was calculated using Equation (1):

(1) $$\mathrm{Encapsulation}\;\mathrm{Efficiency}\;\left(\mathrm{EE}\%\right)=\;\frac{\mathrm{Quantity}\;\mathrm{of}\;\mathrm{Total}\;\mathrm{Peptide}-\mathrm{Quantity}\;\mathrm{of}\;\mathrm{Free}\;\mathrm{Peptide}}{\mathrm{Quantity}\;\mathrm{of}\;\mathrm{Total}\;\mathrm{Peptide}}\times 100$$

### 2.4. Synthesis of the PEG-PCL-PEG Copolymeric Thermogel

A PEG-PCL-PEG copolymer was synthesised by ring-opening polymerisation of ε-caprolactone using MPEG (MW 4000) as a macro-initiator with the inclusion of stannous octate (Sn(Oct)_2_) as a catalyst and IPDI as a coupling agent as described and schematised by Gong et al. [25]. Briefly, 0.098 M ε-caprolactone and 0.007 M of MPEG 4000 were added into a three necked vessel under a dry nitrogen atmosphere. Stannous octate (100 µL) was added and maintained at the reaction temperature of 125 °C for 24 h, with IPDI (0.008 M) added as a coupling agent at the 6 h point with stirring. The resultant copolymer was degassed for 10 min. In order to isolate the pure copolymer, the product was cooled to room temperature, dissolved in dichloromethane and subsequently precipitated with petroleum ether which was previously refrigerated for 1 h. The precipitate was then filtered and vacuum dried at room temperature for 24 h. Pluronic F 127 (PLU) and a mixture of PEG-PCL-PEG/PLU hydrogels were prepared for comparative evaluations in the following concentrations: PLU-10%, 15% and 25% *w*/*v*; PEG-PCL-PEG-10%, 15% and 25% *w*/*v*; and PEG-PCL-PEG/PLU-25% *w*/*v* of PEG-PCL-PEG and 25% *w*/*v* of PLU in ratios of 1:0.5, 1:0.75 and 1:1.

### 2.5. Characterisation of the PEG-PCL-PEG Copolymeric Thermogel

#### 2.5.1. Chemical Structure and Integrity Analysis of the Thermogel System

Fourier-transform infrared (FTIR) spectra of the synthesised copolymer were recorded using a Spectrum 2000 spectrometer with a MIRTGS detector (PerkinElmer Spectrum 100, Wales, UK). Samples were processed by a universal ATR polarisation accessory for the FTIR spectrum series. The resolution was at 4 cm^−1^ and scanned from wavenumber 600 to 4000 cm^−1^. In addition, ^1^H-nuclear magnetic resonance (^1^H-NMR) spectra in CDCl_3_ were generated on a spectrometer (Fourier 300, Bruker, Switzerland) at 400 MHz in order to characterise the integrity of the synthesised copolymer.

#### 2.5.2. Thermosensitive Behaviour of the Nano-Thermogel System

##### Rheological Transitions of the Thermogel

The thermosensitive behaviour of the PEG-PCL-PEG hydrogel and PEG-PCL-PEG/PLU mixture was rheologically analysed. The rheological measurements were investigated employing a Haake MARS, Modular Advanced Rheometer system (Thermoscientific, Dreieich, Germany). The instrument was pre-equipped with a thermo-bath that controlled the temperature of the sample chamber within the required value. In order to measure the gelation temperature of the copolymer solution, temperature sweep analysis was performed at 1 Hz, at a rate of temperature increase of 1 °C/min between 10–60 °C. The dynamic viscoelastic property such as the shear storage modulus (G’) was measured as a function of temperature for different concentrations of the hydrogel formulation.

##### Determination of Sol-Gel Conversion: Tube Inversion Method

It is imperative to study the sol-gel conversion of the hydrogels, as an appropriate gelation temperature and the strength of the injected gel are necessary parameters for in vivo application. Various concentrations of PEG-PCL-PEG and PLU copolymer solutions were prepared and 3 mL of each polymer solution was placed into the 10 mL screw-capped glass vials. The tubes were immersed into a water bath where the temperature was regulated to increase from 20 °C to 60 °C at 2 °C/min. The time and temperature required to affect a sol-gel conversion was visualised by inverting the tube. The physical state at which there was no visual flow of the polymer solution was characterised as the gel state. The experiment was conducted on the native PEG-PCL-PEG and Pluronic hydrogels at different concentrations. Sol-gel conversion of the PEG-PCL-PEG/PLU blend was evaluated at varying ratios of Pluronic and PEG-PCL-PEG copolymers.

#### 2.5.3. Swelling Behaviour of the Thermogel System

For determining the swelling potential of the gel for prediction of in vivo behaviour following ocular injection, 1 mL of the PEG-PCL-PEG, Pluronic and PEG-PCL-PEG/PLU solution was placed into a 10 mL vial, and gelled at 37 °C. The height of the gel was recorded as zero-time. Phosphate buffered saline (PBS, 3 mL, pH 7.4) was added into the vial and incubated at 37 °C at 50rpm. At specified time points, replacement of the medium with 3 mL fresh PBS was performed and the gel height (H_x_) was recorded until 60 days. Calculation of the swelling ratio was as per Equation (2):

(2) $$\mathrm{Swelling}\;\mathrm{Ratio}=\frac{H_{x}-H_{0}}{H_{0}}\times 100$$

where H_0_ and H_x_ are the heights of the gel at zero-time and specified time intervals (i.e., 3 h, 6 h, 12 h, etc.), respectively.

#### 2.5.4. Thermal Transitions of the Thermogel System

Thermal properties of PEG-PCL-PEG, PLU and PEG-PCL-PEG/PLU hydrogels were determined by differential scanning calorimetry (DSC) analysis using STAR^e^ System coupled with analytical computational software (Mettler Toledo, Greifensee, Switzerland). Approximately 10 mg of each polymer was placed in crimped aluminium pans. The melting and re-crystallisation behaviour of the various hydrogel combinations in the range of 0–150 °C with a heating and cooling rate of 5 °C/min was carried out.

#### 2.5.5. Determination of the Surface Architecture of the Thermogel System

Surface morphology of the PEG-PCL-PEG, PLU and PEG-PCL-PEG/PLU dried hydrogels was investigated employing scanning electron microscopy (SEM) (FEI Nova Nanolab 600 FIB/SEM, Hillsboro, OR, USA). Cross-sections of lyophilised hydrogels were mounted on aluminium stubs and gold sputter coated for acquisition of scanning electron microscopic images.

#### 2.5.6. In Vitro Peptide Release from the Nanoparticles and Nano-Enabled Thermogel System

In vitro peptide release kinetics of p11 peptide from PLGA nanoparticles and the nano-thermogel were analysed in PBS (pH 7.4, 37 °C) with 0.02% sodium azide. Briefly, PLGA nanoparticles containing 20 µg p11 peptide were accurately weighed based on the encapsulation efficiency and were re-suspended in 2 mL PBS and transferred into a dialysis bag (MWCO 12KDa). The nanoparticle suspension was then immersed into 10 mL PBS and incubated at 37 °C at 50 rpm. At specific time intervals, 200 µL of PBS was removed, centrifuged and the supernatant analysed for p11 peptide. The pellet was resuspended with 200 µL fresh PBS and added to the vial to maintain sink conditions. For release studies from the nanoparticle-embedded hydrogels, accurately weighed nanoparticles were mixed with respective polymer solutions at <20 °C and transferred into a dialysis bag (molecular weight cut off 12 KDa). The dialysis bag with polymer solutions was immersed into 10 mL sterile PBS, and pre-heated to 37 °C for 2 min for gel formation. The vials were subsequently incubated at 37 °C 50 rpm. At specific time intervals, 200 µL PBS was removed, centrifuged and the supernatant was analysed for p11 peptide. The pellet was resuspended with 200 µL fresh PBS and added to the vial to maintain sink conditions.

The in vitro peptide release data were fitted to various kinetic models in order to describe the release mechanisms. The following plots were constructed: cumulative % drug release vs. time (zero order kinetic model); log cumulative of % drug remaining vs. time (first order kinetic model); cumulative % drug release vs. square root of time (Higuchi model) log cumulative % drug release vs. log time (Korsmeyer model) and cube root of drug % remaining in matrix vs. time (Hixson–Crowell cube root law). The Korsmeyer–Peppas model provides a simple relationship describing drug release from a polymeric system. The *n* value is used to characterise different release mechanisms as for cylindrical shaped matrices, which may be *n* ≤ 0.45 for Fickian diffusion, 0.45 < *n* < 0.89 for anomalous (non-Fickian) diffusion, *n* = 0.89 for case-II transport and *n* > 0.89 for super case-II transport.

Stability of the released peptides was evaluated using 16.5% Tris-Tricine poly acrylamide gel electrophoresis. SDS-PAGE was thus employed to ascertain that the molecular mass or integrity of the released peptide (i.e., stability) was maintained and that it did not undergo extensive proteolysis (protein hydrolysis). The experiment was repeated similarly to the in vitro release study and 20µL of the sample was collected after 5, 10, 20, 35, 45 and 55 days. The 20 µL samples were mixed with 2× Tricine sample buffer (100mM Tris-HCl, pH 6.8, 5% *v*/*v* mercaptoethanol, 0.02% *w*/*v* bromophenol blue, 24% *w*/*v* glycerol, 8% *w*/*v* SDS), heated at 65°C for 2 min and loaded in the well containing 16.5% Tris-Tricine gel. The gel was run under non-reducing conditions at 75 V for 55 min using Tris-Tricine buffer (0.1 M Tris, 0.1M Tricine, 0.1% *w*/*v* SDS pH 8.25). The 16.5% gel was then stained with a Proteosilver silver staining kit (Sigma Aldrich, St Louis, MO, USA) and imaged using Imagequant 300, version 1.0.3, GE Healthcare Lifesciences, Germany.

##### UPLC Analysis for p11 Peptide Determination

The quantitative determination of p11 peptides was carried out employing ultra performance liquid chromatography (UPLC, Waters^®^ Acquity UPLC^TM^ system, Miliford, MA, USA) via adaptation from a method previously developed for UPLC detection of peptides [32]. Gradient baseline separation was obtained with parameters and conditions elaborated in Table 1. Prior filtration of all solutions and solvents was performed through a 0.22 µm filter (Millipore Co. Billerica, MA, USA). The samples for analysis were centrifuged at 20,000 rpm for 5 min at 4 °C.

### 2.6. Biocompatibility Evaluation of the Nano-Enabled Thermogel System

Nano-thermogel biocompatibility was evaluated in a retinal pigment epithelial cell culture (RPE-1, ATCC). Maintenance of the cells was achieved in DMEM, incorporating 10% FBS and penicillin/streptomycin antibiotics at 37 °C, 95% humidity and 5% CO_2_. Sub-culturing of the cells was undertaken every second day employing trypsin-EDTA with a seeding density 5 × 10^4^ cells/mL.

For biocompatibility evaluation, sterile samples (0.5 mL) of each copolymer solution (25% *w*/*v* of PLU, PEG-PCL-PEG, PEG-PCL-PEG/PLU and NP/PLU/PEG-PCL-PEG), achieved by sterile filtration through a 0.22 µm filter (possessing the least impact on rheological properties of hydrogels), were placed into Eppendorf tubes and were incubated at 37 °C to form the gel. FBS (1.5 mL of 10%) containing DMEM was added to the gel in each Eppendorf tube and incubated at 37 °C for 60 days. At each time point (1, 3, 5, 10, 20, 30, 50 and 60 days), 200 µL of the medium was sampled and replaced with fresh DMEM. The sample medium was stored at 4 °C.

A 3-(4,5-dimethylthiazol-2-yl)-2,5- diphenyltetrazolium bromide (MTT) cell viability assay was undertaken to evaluate the cytotoxicity of hydrogels towards RPE-1 cells. Seeding of the cells onto a 96-well plate with a seeding density of 10,000 cells/well was undertaken. After confluency, the medium was removed with addition of 100 µL of release medium and incubation for 24 and 48 h. Following incubation, there was careful removal of the test medium and addition of 10% MTT solution followed by incubation for 3 h at 37 °C and 5% CO_2_. Subsequently, MTT solvent (100 µL) was added with careful mixing, and the absorbance measured at 570 nm.

### 2.7. Haemolytic Activity of the Nano-Enabled Thermogel System

The direct contact method was employed to study the haemolytic activity of the various thermogel combinations (PLU, PEG-PCL-PEG, PEG-PCL-PEG/PLU) and the nano-enabled thermogel (NP/PLU/PEG-PCL-PEG). The application of whole rabbit blood enabled determination of the haemolysis of the samples. Whole blood (450 µL) was treated with 50 µL of hydrogel, agitated gently and incubated at 37 °C for 3 h. Triton × 100 is taken as the positive control and polyethylene glycol as the negative control. Centrifugation of the suspension at 1000 rpm for 15 min was performed following incubation, with subsequent measurement of the absorbance of the supernatant of each tube via a multiplate reader (Bio-Rad, Model 680, UK) at a wavelength of 545 nm. The percentage haemolysis was calculated using Equation (3):

(3) $$\mathrm{Haemolysis}\%=\frac{{\mathrm{OD}}_{S}-\mathrm{ODNC}}{{\mathrm{OD}}_{\mathrm{PC}}-{\mathrm{OD}}_{\mathrm{NC}}}\times 100$$

where OD refers to optical density, S refers to sample, NC is the negative control and PC is the positive control.

### 2.8. Data Presentation and Statistical Analyses

Unless otherwise indicated, data are represented as the mean ± SD. Statistical significance was determined using a Student’s *t*-test with a 95% confidence interval, unless otherwise noted. Statistical calculations were performed employing Microsoft Excel.

## 3. Results

### 3.1. Synthetic Parameters and Characterisation of the PLGA Nanoparticles

**Figure 2:**
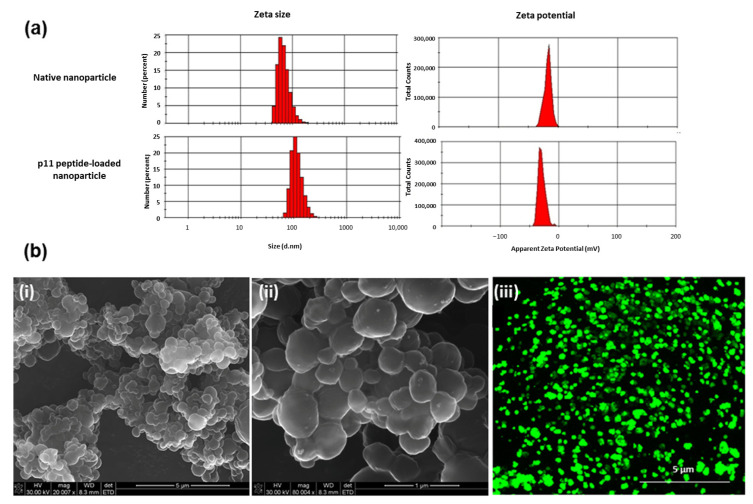
(**a**) Particle size and zeta potential analysis of the native and FITC-p11 peptide-encapsulated nanoparticles. (**b**) (i) SEM (scanning electron microscopy) image of nanoparticle. (ii) High magnification SEM image. (iii) Confocal microscopic image of the FITC-P11 peptide-encapsulated nanoparticles.

In this study, PLGA nanoparticles were prepared for the stable encapsulation of p11 peptide. A double emulsion approach was followed for PLGA nanoparticle formulation. The primary emulsion was prepared by blending PLGA solution in the organic phase with the aqueous phase containing FITC-conjugated peptide molecules. Table 2 describes the parameters adopted for preparing different nanoparticles. In this study, the time of agitation for the second emulsion was varied between 2–10 min while keeping the homogenising speed constant. The particle size, polydispersity index and encapsulation efficiency of the nanoparticles were ascertained (Figure 2a, Table 2). Results showed that particle size varied from 110 nm to 416 nm, with Formulation 1 having the maximum size as compared with Formulation 4. It was observed that as the agitation time of the second emulsion increased, the particle size decreased from 416 nm to 110 nm. This reduction in particle size is a common observation, with the increasing shear rate from the increased stirring speed reducing the droplet size of the emulsion and ultimately the nanoparticles formed, as also observed for example by Geng et al. [31] for their PLGA nanoparticles. However, increasing the stirring time further led to an increase in the particle size, i.e., 220.6 nm for Formulation 5. A similar trend was observed for the polydispersity indices. This higher stirring speed may have destabilised the emulsion leading to enhanced nanoparticle collision and subsequent aggregation with subsequent formation of some larger nanoparticles, and a corresponding increase in the polydispersity index.

The PLGA nanoparticles formulated display a negative zeta potential as a result of the ionised carboxyl groups present in PLGA as reported by various investigators such as Nicolete et al. [33]. Loading of p11 peptide into the nanoparticles increased the negative surface charge of the nanoparticles. The p11 anti-angiogenic peptide possesses regions of positivity and negativity. At the neutral pH at which the zeta potential was measured, an overall negative charge dominated, resulting in the more negative surface charge following peptide encapsulation.

Formulation 1 obtained the highest peptide encapsulation (69.5%); further increases in the agitation to 10 min decreased the efficiency to 14.8%. The results are consistent with previous reports by Geng et al. [31] where various parameters affecting the nanoparticle size and encapsulation efficiency were studied. In terms of encapsulation efficiency, particle size and polydispersity index, Formulation 3 was identified as the preferred system having an average particle size of 128.4nm, with a polydispersity index of 0.219 and an encapsulation efficiency of 63.7%.

SEM images of the optimal PLGA nanoparticles (Formulation 3) revealed a spherical, smooth morphology in the nano-range (Figure 2b (i,ii)). The FITC-p11 peptide encapsulation was confirmed with confocal microscopic images of the nanoparticle suspension at 488 nm excitation (Figure 2b (iii)).

### 3.2. Synthesis and Characterisation of the PEG-PCL-PEG Copolymer Thermogel System

**Figure 3:**
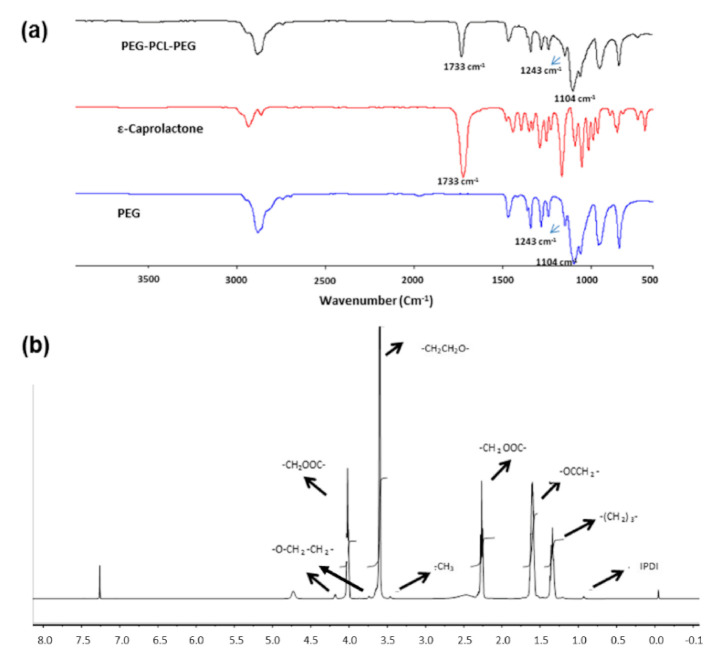
(**a**) Fourier-transform infrared spectroscopy of PEG, ε-caprolactone and PEG-PCL-PEG (polyethylene glycol-polycaprolactone-polyethylene glycol). (**b**) NMR spectroscopy of PEG-PCL-PEG.

Figure 3a presents FTIR spectra of PEG-PCL-PEG triblock copolymer. The characteristic absorption peak of the repeated –OCH_2_CH_2_ unit of PEG and –COO-stretching vibration were observed at 1104 cm^−1^ and 1243 cm^−1^, respectively [18,25]. A solid absorption peak was observed at 1733cm^−1^, representing the stretching vibration of the C=O ester carbonyl group of PCL. The absorption bands at 2943 cm^−1^ and 2868 cm^−1^ correspond to the C–H stretching vibrations of PCL and PEG, respectively. Thus, FTIR spectra showed characteristic peaks of both PEG and PCL segments in the PEG-PCL-PEG copolymer.

The copolymer was further characterised by ^1^H-NMR spectroscopy to confirm the synthesis of PEG-PCL-PEG. The characteristic absorption peaks were indicated in Figure 3b. The sharp absorption peak at 3.6 represents the methylene protons of the –CH_2_CH_2_O-group in the PEG block, while the smaller peak at 3.4 ppm is attributed to the –OCH_3_ end group in the PEG block. The peaks at 1.35, 1.6, 2.32, and 4.06 ppm are attributed to –(CH_2_)_3_–, –OCCH_2_– and –CH_2_OOC– methylene protons in the PCL block. Weak peaks at 4.2 and 3.75 ppm indicate the methylene protons of –O-CH_2_-CH_2_– in the PEG end blocks which link with PCL. The peak at 0.9 ppm represents the –CH_3_ end group of IPDI. This correlates with the observations of Gong et al. [25], who also undertook further extensive characterisation of this triblock [18,26,27]; the PEG-PCL-PEG copolymer formulated herein was achieved via their synthetic approach.

#### 3.2.1. Temperature-Dependent Sol-Gel Transitions of the Thermogel

**Figure 4:**
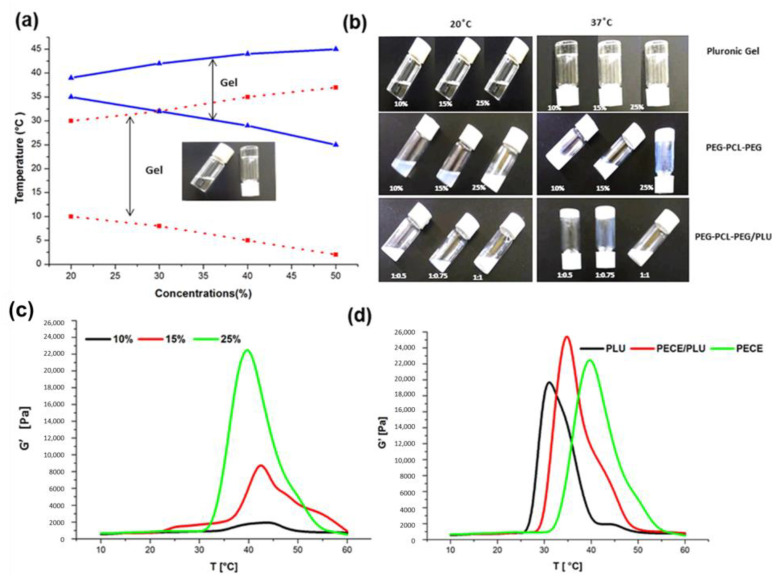
(**a**) Graphical depiction of sol-gel conversion of Pluronic F127 (red) and PEG-PCL-PEG hydrogel (blue). (**b**) Photograph of tube inversion method for sol-gel conversion of the hydrogels at different concentrations. Rheological analysis of (**c**) PEG-PCL-PEG hydrogels at different concentrations and **(d)** comparative analysis of Pluronic, PEG-PCL-PEG and PEG-PCL-PEG/PLU hydrogels at a concentration of 25% *w*/*v*.

The sol-gel conversion of PEG-PCL-PEG in comparison to Pluronic (PLU) thermogel at different concentrations was studied. In comparison to Pluronic, PEG-PCL-PEG has a very low sol-gel conversion window. It was observed that below a 10% *w*/*v* concentration, PEG-PCL-PEG could not form a gel over the temperature range of 0–50 °C. Aqueous solutions of PEG-PCL-PEG transformed into a gel at a concentration above the critical gelation concentration as the temperature (lower critical gelation temperature, LCGT) increased. This is primarily due to micelle packing and aggregation [34,35]. Sol-gel conversion of 20% *w*/*v* PEG-PCL-PEG was transient with a very narrow conversion window i.e., between 36 °C and 40 °C. Above 40 °C, the gel rapidly transformed into its solution form. Further, as the concentration increased the sol-gel conversion window broadened, with a shift in LCGT from 35 °C to 24 °C as concentration increased to 20% and 50% *w*/*v*, respectively (Figure 4a). Moreover, the upper critical gelation temperature (UCGT) shifted from 38 °C to 45 °C as concentration varied from 20 to 50% *w*/*v*. Pluronic hydrogel showed a broad conversion window starting from the concentration of 10% *w*/*v* (Figure 4a). The LCGT of Pluronic gel was observed as 10 °C–0 °C for 20–50% *w*/*v* and UCGT as 29 °C and 35 °C for the concentration 20% *w*/*v* and 50% *w*/*v*, respectively.

Sol-gel conversion of PEG-PCL-PEG/PLU hydrogel solution was carried out using 25% *w*/*v* of both the polymer solutions at varying ratios. Three different ratios of PEG-PCL-PEG/PLU were analysed viz., 1:0.5, 1:0.75 and 1:1. It was observed that the 1:1 concentration, PEG-PCL-PEG/PLU could not form a gel at 37 °C compared to 1:0.5 and 1:0.75 ratios (Figure 4b).

The effect of the addition of PLGA nanoparticles on the systems was that they slightly reduced the sol-gel transition temperature of the hydrogels as the NPs expanded the temperature range of the sol phase compared to the pristine networks, but this effect was found to be minimal during preliminary investigations (*p* > 0.05).

These observations were further analysed via rheological analysis (Figure 4c,d). The investigation on modulus of the gel (G’) vs. temperature for varying concentrations of PEG-PCL-PEG and a comparative study on PLU, PEG-PCL-PEG and PEG-PCL-PEG/PLU (1:0.5) were carried out. Generally, sol-gel transition occurs with a notable variation in modulus [35,36]. Figure 4c shows that as the concentration of the PEG-PCL-PEG increased the modulus also increased. There is no significant difference in modulus in the case of PLU and PEG-PCL-PEG with similar concentrations (25% *w*/*v*). Blending with PLU increases the modulus of PEG-PCL-PEG compared to the individual components.

#### 3.2.2. Thermal Analysis of the Thermogel System

**Figure 5:**
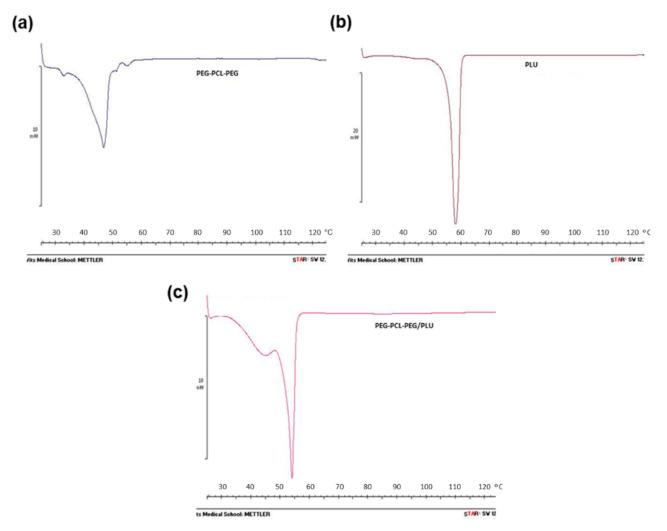
DSC (differential scanning calorimetry) thermograms of synthesised and lyophilised hydrogels (**a**) PEG-PCL-PEG, (**b**) Pluronic and (**c**) PEG-PCL-PEG/PLU.

Thermograms of optimised PEG-PCL-PEG (25% *w*/*v*), PLU (25% *w*/*v*) and PEG-PCL-PEG/PLU (25% *w*/*v* of each polymer in 1:0.5 ratio) after lyophilisation were also investigated (Figure 5a–c). The melting peak observed for PLU was at 60 °C. PEG-PCL-PEG displayed two endothermic melting transitions at 47 °C and 32 °C, representative of the melting points of the polymeric components of the triblock, and congruent with the observations of Gong et al. [18]. However, the blending of the PEG-PCL-PEG with PLU shows a shift in the melting points of all polymers to a slightly lower temperatures (55, 44 and 26 °C) as compared to PLU and PEG-PCL-PEG alone.

#### 3.2.3. Swelling Behaviour of the Thermogel System

**Figure 6:**
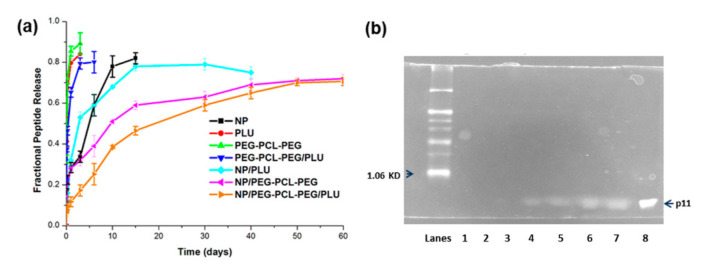
Graphical depiction of swelling ratio of the investigated hydrogel systems.

The developed PEG-PCL-PEG/PLU hydrogel was incubated in PBS for 60 days in order to investigate the swelling characteristics of the gel in order to ascertain whether there would be any potential pressure effects on the ocular tissues following injection, resulting in potential tissue damage. Figure 6 highlights that PEG-PCL-PEG and PEG-PCL-PEG/PLU gel did not show significant change (*p* > 0.05) in the height of the gel even after 60 days of contact with PBS, as compared to pure PLU. At the initial stage (~5 days) a trivial increase in the height was observed, thereafter the height did not vary notably over the 60 days of measurement, and thus any negative pressure effects on surrounding ocular tissues following injection would proposedly be minimal.

### 3.3. In Vitro Peptide Release from the Nanoparticles and Nano-Enabled Thermogel System

**Figure 7:**
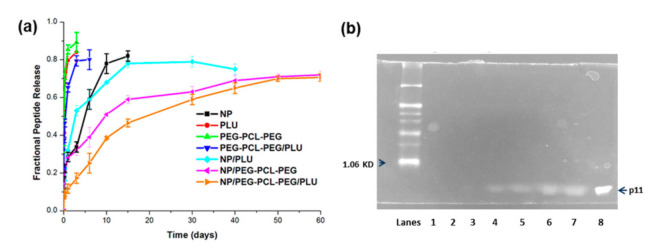
(**a**) Plot depicting the in vitro p11 peptide release from PLGA nanoparticles, Pluronic hydrogel, PLU/PEG-PCL-PEG hydrogel, PEG-PCL-PEG hydrogel, nanoparticle-loaded PEG-PCL-PEG hydrogel and nanoparticle-loaded PEG-PCL-PEG/PLU hydrogel at 37 °C, in PBS for a period of 60 days. (**b**) SDS–PAGE results of p11 peptide in vitro release profile (Lane 1, marker; Lane 2, 5days; Lane 3, 10 days; Lane 4, 20 days; Lane 5, 35 days; Lane 6, 45 days; Lane 7, 55 days; Lane 8 control p11 peptide.

The in vitro release behaviour of p11 peptide was undertaken in PBS (pH 7.4, 37 °C) for a period of 60 days as graphically depicted in Figure 7a. In this study, 50% of the peptide was released within 5 h from the PLU, PEG-PCL-PEG and PEG-PCL-PEG/PLU hydrogel system, whereas there was a controlled release of 75–80% of peptide from the PLGA nanoparticles over a period of 10–15 days with an initial burst release of 33.6 ± 2.8% in 3 days. The nano-enabled PEG-PCL-PEG hydrogel system demonstrated a sustained release of 70.6 ± 1.98% peptide over 60 days. However, peptide release from nanoparticles (NP) embedded in the PEG-PCL-PEG/PLU hydrogel was much slower in comparison to NP embedded in PEG-PCL-PEG. It was observed that 50% of peptide release from NP/PEG-PCL-PEG and NP/PEG-PCL-PEG/PLU was at 9.5 and 19.2 days, respectively. In the case of NP/PLU hydrogel, 50% peptide release occurred before the 5th day of incubation.

Peptide release from the NPs was best described by the Higuchi model (R^2^ = 0.948), indicating release of peptide from the NP matrix as a square root of time-dependent process based on Fickian diffusion. The Higuchi model also described release of the peptide from PLU, PEG-PCL-PEG and PEG-PCL-PEG (R^2^ = 0.9201, R^2^ = 0.9267 and R^2^ = 0.8482, respectively). Following incorporation of the NPs into the hydrogels, peptide release followed the Higuchi model for NP/PLU (R^2^ = 0.8993) and the Korsmeyer–Peppas model for NP/PEG-PCL-PEG and NP/PEG-PCL-PEG/PLU (R^2^ = 0.9615 and R^2^ = 0.9778, respectively). When examining the release mechanism in accordance with the *n* value of the Korsmeyer–Peppas model, Fickian release was the dominant mechanism for both the NP/PLU system (*n* = 0.3294) and the NP/PEG-PCL-PEG system (*n* = 0.221), whereas an anomalous/non-Fickian diffusion was observed from the NP/PEG-PCL-PEG/PLU system (*n* = 0.4711) indicating a combination of both diffusion of peptide through the polymer and ultimate dissolution/erosion of the polymer.

Further, the stability/integrity of the released peptide was confirmed using Tris-Tricine polyacrylamide gel electrophoresis. According to Figure 7b, the band for the control p11 peptide (Lane 8) with a molecular weight of 721 Da appeared below the lowest protein marker position 1060 Da (Lane 1). The band for all p11 peptides released from the 5th day to 55th day can be visualised at the same level from Lane 2–7. Lane 2 and 3 showed very faint bands because of the lower amount of peptide released at the 5th and 10th day. From these bands it is clearly evident that the protein released was stable even after 55 days of incubation.

### 3.4. Biocompatibility Evaluation of the Nano-Enabled Thermogel System

**Figure 8:**
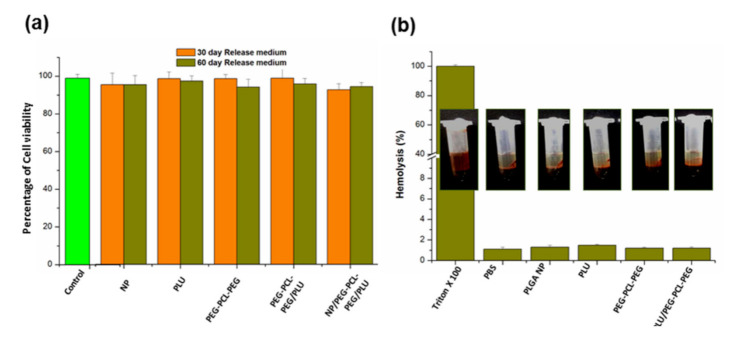
(**a**) Assessment of percentage cell viability after incubation with 30 days and 60 days release medium using MTT assay. (**b**) Analysis of haemolytic potential of different hydrogel systems and PLGA nanoparticles. Inset: Representative photograph showing no significant haemolysis in the hydrogel systems and PLGA nanoparticles in comparison to positive control (Triton × 100).

The biocompatibility of PLGA nanoparticles, 25% *w*/*v* of PLU, PEG-PCL-PEG, PEG-PCL-PEG/PLU and NP/PEG-PCL-PEG/PLU hydrogel was investigated using retinal pigment epithelial cells (RPE-1). An indirect toxicity study was carried out via MTT assay. The release media of all the different formulations after 30 days and 60 days were analysed. After 24 h of incubation with the released medium, no significant cell toxicity was observed (*p* > 0.05). The viability in the control well was recorded as 98.9 ± 2.1%. In the case of all the formulations studied, the cell viability was in the range of 94–98%, which confirms the biocompatibility of the hydrogel system (Figure 8a).

### 3.5. Haemolytic Activity of the Nano-Enabled Thermogel System

A red blood cell haemolysis assay was performed to determine the intensity of ocular irritation caused by the developed NP/PEG-PCL-PEG/PLU as well as the individual components, namely NP alone, PLU, PEG-PCL-PEG and PEG-PCL-PEG/PLU. It was evident from Figure 8b that the nanoparticles alone as well as other hydrogel systems did not evoke significant levels of haemolysis as compared to Triton × 100, which gave approximately 100% haemolysis. Polyethylene glycol was used as the negative control and showed 1.1 ± 0.99% haemolysis which is comparable with all the samples analysed tested (1.2–1.5%). As per the reported criteria for blood damage provided in the ASTM E2524-08 standard, a percentage of haemolysis <5% was considered as normal [37].

## 4. Discussion

Since the eye is a sequestered tissue compartment, the direct delivery of therapeutic agents is always advantageous over systemic administration with regards to the bioavailability and side effects [38]. Even though intravitreal injections of anti-VEGF molecules can circumvent most of the disadvantages of other administration routes, for angiogenesis-related posterior segment ocular afflictions, repeated weekly injections could potentially instigate the development of threatening complications such as haemorrhage and retinal detachment, and eventually cause blindness [39]. Moreover, since VEGF is a survival factor of normal newly formed retinal vessels, the targeting of VEGF will affect their normal growth [11]. This study focused on an anti-angiogenic hexapeptide, having the PDZ binding motif (Ser-Asp-Val) possessing high affinity to alpha v beta 3 (α_v_β_3_) integrin molecules whose anti-angiogenic effect has been already studied and established using human endothelial cells. Bang et al. [14] observed that the peptide can easily be taken up by the endothelial cells via α_v_β_3_ integrins and resulted in a bFGF-mediated suppression of endothelial cell proliferation through mitogen-activated protein kinase in addition to p53-mediated apoptosis associated with caspase activation.

FTIR spectra of the PEG-PCL-PEG copolymer revealed all the characteristic peaks of PEG (1104 cm^−1^ and 1243 cm^−1^) and PCL (1733 cm^−1^) components [18,25,26]. Consistent with previous reports by Gong et al. [18,25], ^1^H-NMR spectroscopy confirmed the formation of PEG-PCL-PEG triblock polymer.

It has been previously highlighted that the PEG-PCL-PEG hydrogel demonstrates thermosensitivity and biodegradability and could be employed as an organic solvent-free injectable controlled drug delivery system [18,25,26,27]. Temperature-dependent sol-gel conversion of the native PEG-PCL-PEG and a blend of PEG-PCL-PEG/Pluronic were studied in comparison to the Pluronic hydrogel using the tube inversion method and rheological studies [40]. The mechanism of gel formation for Pluronic as well as PEG-PCL-PEG hydrogel has been previously reported [41]. As the concentration of the polymer increased, the time required for gelation decreased. In this study, 25% PEG-PCL-PEG was selected as the preferred concentration based on the ease of preparation. Incorporation of 25% PLU with PEG-PCL-PEG further increased the modulus of the system and decreased the time required for gelation. Sol-gel conversion windows of 29 °C–45 °C and 32 °C–47 °C were observed in the case of PEG-PCL-PEG and PEG-PCL-PEG/PLU, respectively, in comparison to the lower sol-gel transition window (26 °C–39 °C) for PLU. The results confirmed the feasibility of the PEG-PCL-PEG-based hydrogel for in vivo application.

The PLGA nanoparticle possessing an average particle size 128.4 nm and an encapsulation efficiency of 63.7% was identified as most favourable for incorporation within the thermogel. Further in vitro release studies of PLGA nanoparticles showed a controlled release of p11 peptide (80%) over a period of 15 days. However, NP-embedded PEG-PCL-PEG and PEG-PCL-PEG/PLU achieved further enhanced sustained release of the peptide from PLGA nanoparticles (70% over a period of 60 days) in comparison to PLU. The Korsmeyer–Peppas model best described drug release from the NP/PEG-PCL-PEG and NP/PEG-PCL-PEG/PLU system. The Korsmeyer–Peppas model is also known as the “Power law”, describing drug release from a polymeric system, and describes some release mechanisms simultaneously, such as the diffusion of water into the matrix, swelling of the matrix and dissolution of the matrix.

These results are in agreement with swelling studies as well as rheological analysis for all the thermogels. Results from swelling studies revealed no significant difference in the swelling ratio between PEG-PCL-PEG and PEG-PCL-PEG/PLU thermogels analysed, which indicates the stability of the hydrogel for a prolonged duration. Pluronic gel showed more rapid swelling. The PEG-PCL-PEG/PLU thermogel possessed the highest G’, thus resulting in a “stiffer” matrix, which further reduced diffusion of nanoparticles and peptide from the system. Thus, release of nanoparticles as well as peptide from the hydrogel system is proposedly primarily due to diffusion through the gel matrix, with minimal swelling and ultimate erosion of the hydrogel, which was confirmed by the kinetic models and release mechanisms described. In addition, the rate of hydrolytic degradation of PCL is reduced [26], which further supports diffusion mediated release. The temperature-dependent degradation profile was studied by Gong et al. [18] who reported that approximately 43% polymer degradation occurred over a period of 49 days. During swelling studies, it was reported that the height of the gel increased and then decreased to zero, which indicated the complete degradation of the gel [18]. In contrast, the gel system reported herein did not show notable fluctuations in gel height, indicating a very low swelling ratio that in turn led to the sustained release of peptide from the system for a period of 60 days. There are various factors determining the release of matrix-embedded drugs. However, the drug liberation and diffusion from a matrix into the already diffused medium present in the matrix and the transport of a drug from the matrix medium into the external medium are the two major determinants of drug release kinetics. Conversely, the scenario is very different from the nano-enabled hydrogel system, where both the nanoparticle wall and the carrier gel matrix impede the release of drug. Initial peptide release would purportedly be from the outer surface of the hydrogel. In this study it was found that up until the 5th day, the amount of peptide released from NP/PEG-PCL-PEG was more or less similar to that from the nanoparticle alone, which could be due to the nanoparticle release from the outer surface of hydrogel. Thereafter, the release rate decreased due to the matrix effect. In addition, more sustained release of peptides from the NP/PEG-PCL-PEG/PLU hydrogel is attributed to its more viscous nature in comparison to NP/PEG-PCL-PEG.

With reference to AMD treatment with anti-VEGF therapy, referring to aflibercept (Eylea™), initial injection frequency is every 4 weeks for 3 months. After three once-monthly loading doses, maintenance doses may be administered every 2 months. After 1 year of effective therapy, patients may be treated with one dose every 12 weeks. With regard to treatment with ranibizumab (Lucentis™), treatment involves three once-monthly loading doses and maintenance doses may be administered every 1 month [7]. No comparator therapies exist on the market for the delivery of p11 peptide for ophthalmic applications; however, as the nano-enabled PEG-PCL-PEG hydrogel system demonstrated a sustained release of 70.6 ± 1.98% peptide over 60 days (2 months), the system could be a potential alternative regimen to the regimens discussed for anti-VEGF therapies.

Gong et al. [27] evaluated the acute toxicity of the PEG-PCL-PEG hydrogel using BALB/c mice following intrapleural, intraperitoneal or subcutaneous administration and reported a lack of toxic response and histopathological changes. The in vivo gel formation and degradation were also evaluated following intraperitoneal and subcutaneous administration. Further, Peng et al. [30] evaluated the ophthalmic potential of the PEG-PCL-PEG hydrogel for application following glaucoma filtration surgery. Their intracameral injection-implanted drug carrier was developed for inhibition of the formation of postoperative scarring and evaluated in a rabbit eye model. They reported a lack of corneal abnormalities and other ocular tissue damage, while sustained release of the anti-VEGF agent inhibited neovascularisation and scar formation. Overall, this highlighted the potential safety of the hydrogel for application in the biomedical field, specifically in ophthalmic applications. In this investigation, biocompatibility and haemolysis studies further confirmed the suitability of the hydrogel system for maintaining the stability and providing sustained release of the anti-angiogenic peptide, thereby improving the therapeutic effect. This study thus provides a proof-of-concept of the anti-angiogenic effect of the developed injectable hydrogel system for the potential treatment of retinal diseases.

## 5. Conclusions

In the present study, PLGA nanoparticles embedded within a PEG-PCL-PEG/PLU thermosensitive hydrogel (nano-thermogel) system were synthesised and employed for the stable and sustained delivery of an anti-angiogenic p11 hexapeptide. The optimised PEG-PCL-PEG/PLU hydrogel was prepared and displayed a sol-gel transition at body temperature. The ability of the hydrogel system to exist as a solution at lower temperatures is advantageous for the preparation of protein/peptide-incorporated injectable systems for in vivo applications. The hydrogel showed successful delivery of stable peptide in a sustained manner when investigated over a period of 60 days. Non-cytotoxicity and non-haemolytic behaviour of the hydrogel was predictive of the in vivo applicability. Moreover, our results suggest that PLGA nanoparticles incorporated within a PEG-PCL-PEG/PLU in situ forming gel are a promising candidate for sustained delivery of p11 peptide for treating various angiogenic-related retinal diseases, as well as tumours. Future investigation in a New Zealand albino rabbit model is required for establishment of the preclinical potential of the p11 peptide and the nano-thermogel system.

## Figures and Tables

**Table 1:** UPLC (ultra performance liquid chromatography) specifications and method parameters.

Parameters	Values/Conditions
Strong wash	90% *v*/*v* acetronitrile 10% *v*/*v* water
Weak wash	10% *v*/*v* water: 90% *v*/*v* acetronitrile
Mobile phase	A: 0.1% TFA * water, B: 0.1% TFA * acetronitrile
Gradient	0% B in 2 min, 0–30% B in 10 min30–90% B in 2 min
Flow rate	1 mL/min
Column specification	C18
Injection volume	7 µL
Run time	12 min
Column temperature	25 °C
Sample temperature	25 °C
Pump pressure	6000 ± 500 psi
Delta value	<10
Wavelength of absorption	220 nm

* Trifluoracetic acid.

**Table 2:** Physicochemical characterisation of p11 peptide-loaded PLGA (poly(lactic-*co*-glycolic acid)) nanoparticles.

Formulation #	Stirring Time (Min)		Zeta Size (nm)	Polydispersity Index	Zeta Potential (mV)	Encapsulation Efficiency (%)
	S1	S2				
F 1	3	2	416.0	0.503	−33.5 ± 4.8	69.5
F 2	3	3	318.4	0.410	−30.3 ± 3.9	65.7
F 3	3	5	128.4	0.219	−34.6 ± 9.1	63.7
F 4	3	8	110.0	0.260	−38.5 ± 4.8	34.9
F 5	3	10	220.6	0.560	−22.5 ± 6.4	14.8

## Data Availability

The data presented in this study is available in the article.

## References

[1] Steinkuller P.G., Du L., Gilbert C., Foster A., Collins M.L., Coats D.K. (1999). Childhood blindness. J. Am. Assoc. Pediat. Ophthalmol. Strabismus.

[2] Rahmani B., Tielsch J.M., Katz J., Gottsch J., Quigley H., Javitt J., Sommer A. (1996). The cause-specific prevalence of visual impairment in an urban population. The Baltimore Eye Survey. Ophthalmology.

[3] Bressler N.M., Bressler S.B. (1995). Preventative ophthalmology. Age-related macular degeneration. Ophthalmology.

[4] Anderson O.A., Bainbridge J.W., Shima D.T. (2010). Delivery of anti-angiogenic molecular therapies for retinal disease. Drug Discov. Today.

[5] Kim L.A., D’Amore P.A. (2012). A brief history of anti-VEGF for the treatment of ocular angiogenesis. Am. J. Pathol..

[6] Hynes R.O. (1987). Integrins: A family of cell surface receptors. Cell.

[7] Prall R.F., Dahl A.A. (2019). Exudative (Wet) Age-Related Macular Degeneration (AMD) Medication, Drugs & Diseases: Ophthalmology. https://emedicine.medscape.com/article/1226030-medication.

[8] Nishijima K., Ng Y.S., Zhong L., Bradley J., Schubert W., Jo N., Akita J., Samuelsson S.J., Robinson G.S., Adamis A.P. (2007). Vascular endothelial growth factor-A is a survival factor for retinal neurons and a critical neuroprotectant during the adaptive response to ischemic injury. Am. J. Pathol..

[9] Saint-Geniez M., Maharaj A.S., Walshe T.E., Tucker B.A., Sekiyama E., Kurihara T., Darland D.C., Young M.J., D’Amore P.A. (2008). Endogenous VEGF is required for visual function: Evidence for a survival role on Müller cells and photoreceptors. PLoS ONE.

[10] Gerhardinger C., Brown L.F., Roy S., Mizutani M., Zucker C.L., Lorenzi M. (1998). Expression of vascular endothelial growth factor in the human retina and in nonproliferative diabetic retinopathy. Am. J. Pathol..

[11] Alon T., Hemo I., Itin A., Pe’er J., Stone J., Keshet E. (1995). Vascular endothelial growth factor acts as a survival factor for newly formed retinal vessels and has implications for retinopathy of prematurity. Nat. Med..

[12] Brooks P.C., Clark R.A., Cheresh D.A. (1994). Requirement of vascular integrin alpha v beta 3 for angiogenesis. Science.

[13] Friedlander M., Theesfeld C.L., Sugita M., Fruttiger M., Thomas M.A., Chang S., Cheresh D.A. (1996). Involvement of integrins alpha v beta 3 and alpha v beta 5 in ocular neovascular diseases. Proc. Natl. Acad. Sci. USA.

[14] Bang J.-Y., Kim E.-Y., Kang D.-K., Chang S.-I., Han M.-H., Bae K.-H., Kang I.-C. (2011). Pharmacoproteomic analysis of a Novel Cell-Permeable Peptide inhibitor of Tumor induced angiogenesis. Mol. Cell Proteome.

[15] Das S., Suresh P.K. (2010). Drug delivery to eye: Special reference to nanoparticles. Int. J. Drug Deliv..

[16] Nguyen Q.D., Shah S.M., Hafiz G., Quinlan E., Sung J., Chu K., Cedarbaum J.M., Campchiaro P.A. (2006). A phase I trial of an IV-administered vascular endothelial growth factor trap for treatment in patients with choroidal neovascularization due to age-related macular degeneration. Ophthalmology.

[17] Prasad A., Schadlu R., Apte R.S. (2007). Intravitreal pharmacotherapy: Applications in retinal disease. Compr. Ophthalmol. Update.

[18] Gong C.Y., Shi S., Dong P.W., Kan B., Gou M.L., Wang X.H., Li X.Y., Luo F., Zhao X., Wei Y.Q. (2009). Synthesis and Characterisation of PEG-PCL-PEG thermosensitive hydrogel. Int. J. Pharm..

[19] Lin G., Cosimbescu L., Karin N.J., Gutowskab A., Tarasevich B.J. (2013). Injectable and thermogelling hydrogels of PCL-g-PEG:mechanisms, rheological and enzymatic degradation properties. J. Mater. Chem. B.

[20] Li F., Hurley B., Liu Y., Leonard B., Griffith M. (2012). Controlled release of bevacizumab through nanospheres for extended treatment of age-related macular degeneration. Open Ophthalmol. J..

[21] Shelke N.B., Kadam R., Tyagi P., Rao V.R., Kompella U.B. (2011). Intravitreal poly(L-lactide) microparticles sustain retinal and choroidal delivery of TG-0054, a hydrophilic drug intended for neovascular diseases. Drug Deliv. Transl. Res..

[22] Short B.G. (2008). Safety evaluation of ocular drug delivery formulations: Techniques and practical considerations. Toxicol. Pathol..

[23] Gao H., Yang Y.W., Fan Y.G., Ma J.B. (2006). Conjugates of poly(DL-lactic acid) with ethylenediamino or diethylenetriamino bridged bis(beta-cyclodextrin)s and their nanoparticles as protein delivery systems. J. Control Release.

[24] Lee S.H., Zhang Z., Feng S.S. (2007). Nanoparticles of poly(lactide)-tocopheryl polyethylene glycol succinate (PLA-TPGS) copolymers for protein drug delivery. Biomaterials.

[25] Gong C.Y., Qian Z.Y., Liu C.B., Huang M.J., Gu Y.C., Wen Y.J., Kan B., Wang K., Dai M., Li X.Y. (2007). A thermosensitive hydrogel based on biodegradable amphiphilic poly (ethylene glycol)–polycaprolactone–poly(ethylene glycol) block copolymers. Smart Mater. Struct..

[26] Gong C.Y., Dong P.W., Shi S., Fu S.Z., Yang J.L., Guo G., Zhao X., Wei Y.Q., Qian Z.Y. (2009). Thermosensitive PEG-PCL-PEG hydrogel controlled drug delivery system: Sol-Gel-Sol transition and in vitro drug release study. J. Pharm. Sci..

[27] Gong C.Y., Wu Q.J., Dong P.W., Shi S., Fu S.Z., Guo G., Hu H.Z., Zhao X., Wei Y.Q., Qian Z.Y. (2009). Acute toxicity evaluation of biodegradable in situ gel-forming controlled drug delivery system based on thermosensitive PEG-PCL-PEG hydrogel. J. Biomed. Mater. Res. B Appl. Biomater..

[28] Cuong N.-V., Hsieh M.-F., Chen Y.-T., Liau I. (2010). Synthesis and Characterization of PEG–PCL–PEG Triblock Copolymers as Carriers of Doxorubicin for the Treatment of Breast Cancer. J. Appl. Polym. Sci..

[29] Azouz H., Dahmoune F., Rezgui F., G’ Sell (2016). Full factorial design optimization of anti-inflammatory drug release by PCL–PEG–PCL microspheres. Mat. Sci. Eng. C.

[30] Peng R., Qin G., Li X., Lv H., Qian Z., Yu L. (2014). The PEG-PCL-PEG Hydrogel as an Implanted Ophthalmic Delivery System after Glaucoma Filtration Surgery; a Pilot Study. Med. Hypothesis Discov. Innov. Ophthalmol..

[31] Geng H., Song H., Qi J., Cui D. (2011). Sustained release of VEGF from PLGA nanoparticles embedded thermosensitive hydrogel in full-thickness porcine bladder acellular matrix. Nanoscale Res. Lett..

[32] Adeyemi S.A. (2017). A Novel Peptide-Enhanced Drug Delivery System for Squamous Cell Oesophageal Carcinoma. Ph.D. Thesis.

[33] Nicolete R., dos Santos D.F., Faccioli L.H. (2011). The uptake of PLGA micro or nanoparticles by macrophages provokes distinct *in vitro* inflammatory response. Int. Immunopharm..

[34] Hwang M.J., Suh J.M., Bae Y.H., Kim S.W., Jeong B. (2005). Caprolactonic poloxamer analog: PEG-PCLPEG. Biomacromolecules.

[35] Bea S.J., Suh J.M., Sohn Y.S., Bae Y.H., Kim S.W., Jeong B. (2005). Thermogelling poly(caprolactone-b-ethylene glycol-b- caprolactone) aqueous solutions. Macromolecules.

[36] Kim I.Y., Yoo M.K., Kim B.C., Park I.Y., Lee H.C., Chou C.S. (2008). Thermogelling behaviors of poly(caprolactone-*b*-ethylene glycol-*b*-caprolactone) triblock copolymer in the presence of hyaluronic acid. J. Polym. Sci. Polym. Chem..

[37] Choi J., Reipa V., Hitchins V.M., Georring P.L., Malinauskas R.A. (2011). Physico chemical characterization and in vitro hemolysis evaluation of silver nanoparticles. Toxicol. Sci..

[38] Thrimawithana T.R., Young S., Bunt C.R., Green C., Alany R.G. (2011). Drug delivery to posterior segment of the eye. Drug Discov. Today.

[39] Geroski D.H., Edelhauser H.F. (2000). Drug delivery for posterior segment eye disease. IOVS.

[40] Patel A.R., Kulkarni S., Nandekar T.D., Vavia P.R. (2008). Evaluation of alkyl polyglucoside as an alternative surfactant in the preparation of peptide-loaded nanoparticles. J. Microencapsul..

[41] Teles H., Vermonden T., Eggink G., Hennink W.E., de Wolf F.A. (2010). Hydrogels of collagen-inspired telechelic triblock copolymers for the sustained release of proteins. J. Control Release.

